# Hydromechanical impact of basement rock on injection-induced seismicity in Illinois Basin

**DOI:** 10.1038/s41598-022-19775-4

**Published:** 2022-09-19

**Authors:** Nikita Bondarenko, Yury Podladchikov, Roman Makhnenko

**Affiliations:** 1 Department of Civil and Environmental Engineering, University of Illinois at Urbana-Champaign, Urbana, IL USA; 2 Institute of Earth Sciences, University of Lausanne, Lausanne, Switzerland

**Keywords:** Seismology, Geophysics, Petrology

## Abstract

The common explanation of observed injection-induced microseismicity is based on the measured stress state at the injection interval and the assumption that it remains the same in the vicinity. We argue here that representing the stress state in different geologic formations over the injection site with the single Mohr’s circle is insufficient due to local stratigraphic features and contrast in compressibilities of the involved formations. The role of hydromechanical coupling in the microseismic response is also crucial for the proper assessment of the problem. Thoroughly monitored Illinois Basin Decatur Project revealed the majority of CO_2_ injection-associated microseismic events being originated in the crystalline basement. Even though basement faults can serve as the conduits for fluid flow—the predicted pressure increase seems to be insufficient to trigger seismicity. To address this issue, accurate laboratory measurements of rock properties from the involved formations are conducted. The pre-injection stress state and its evolution are evaluated with the hydromechanically coupled numerical model. It appears that the presence of an offset in a stiff competent layer affects the stress state in its vicinity. Therefore, both the pre-injection stress state and its evolution during the fluid injection should be addressed during the induced seismicity assessment.

## Introduction

Human-induced seismicity^1^ and injection-induced seismicity in particular^2^ is a well-recognized hazard during many industrial activities. Despite previous extensive studies of this issue, the unified approach to assess induced seismicity potential during geoenergy applications has not yet been developed^3^. While the storage of carbon dioxide (CO_2_) in geologic formations (e.g., deep saline aquifers) is considered as an essential part of many climate change mitigation pathways^4^, the possibility of large magnitude earthquakes during CO_2_ injection remains a debatable topic^5–7^. At this moment, only microseismicity imperceptible on the surface is recorded during the demonstration-scale injections of CO_2_^8^. Successful implementation of pilot-scale projects significantly impacts public perception and is required for further scaling of the injection volume. These projects are usually well-instrumented and an intensive monitoring program is implemented to guarantee their success. The monitoring activities include continuous subsurface measurements (pressure recording, microseismic activity monitoring, repeating logging measurements, and seismic surveys), groundwater control, as well as atmospheric and InSAR monitoring. In addition, intensive pre-injection site characterization (including the laboratory testing of the extracted rock) is conducted. Therefore, data collected during carefully investigated pilot-scale projects possess a great interest to improve the understanding of the mechanisms responsible for features of induced seismicity.

Particular attention should be given to the assessment of induced seismicity risks in the crystalline basement rock underlying the injection interval since the majority of observed events have occurred below the reservoir^9^. In contrast to the reservoir rock, the stress state typical for crystalline rock is more favorable for reactivation of pre-existing faults^5^, and high-permeable faults might serve as pathways for pore pressure migration into low-permeable crystalline rock^10^. For example, despite comprehensive instrumentation and intensive pre-injection site assessment, the mechanism for the injection-induced microseismicity observed during CO_2_ injection in Illinois Basin remains to be unclear^11^. The widely utilized approach based on fault reactivation due to changes solely in the pore pressure requires high overpressure to reach failure. This mechanism is not capable of explaining the observed microseismicity in Illinois Basin with recorded fluid overpressure on the order of megapascals^12,13^. Even if strength parameters for the in-situ material are lower than values measured in the laboratory due to the presence of fractures and faults—it is unlikely that the friction angle of rock is significantly affected by it if a large amount of the infilling material is not involved. It is expected that more advanced models are needed to address the coupling between fluid flow and mechanical behavior, poroelastic stressing effect, and geologic features of stratigraphy, among others^14–16^. These phenomena might be responsible for microseismicity observed at stresses lower than those predicted by the simplified models and explain an absence of clear correlation between the propagation of pressure front and clustering of the microseismic events.

**Figure 1:**
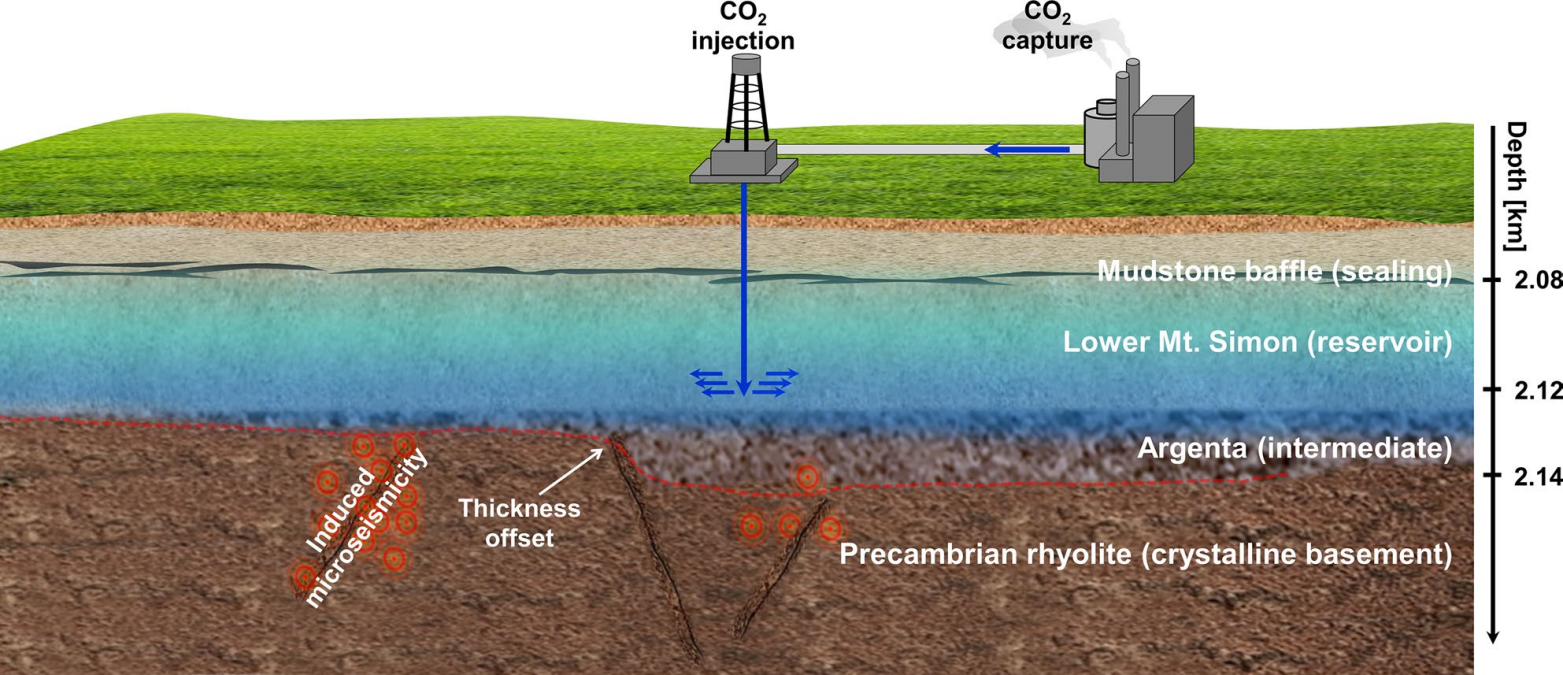
Sketch of stratigraphy involved in CO_2_ injection into the deep saline aquifer in Illinois Basin. Low-permeable mudstone baffles restrict the vertical flow of CO_2_ to the upper part of the reservoir. The thickness of the intermediate layer (Argenta) abruptly thins over topographic high in the crystalline basement (Precambrian rhyolite). Observed induced microseismicity is likely to be associated with the reactivation of the critically stressed basement faults.

The Illinois Basin Decatur Project (IBDP) involved injecting approximately 1 million metric tons of CO_2_ into the reservoir formation over 3 years^11^. The injection interval is located in the lower part of Mt. Simon sandstone near the crystalline basement represented by Precambrian rhyolite (Fig. 1). Utilization of the waveform cross-correlation approach results in a depth resolution of located injection-induced microseismic events on the order of tens of meters and associates these events with the crystalline basement below the injection interval^17^. The feature of the Lower unit of Mt. Simon sandstone is the presence of a low permeable intermediate basal sealing layer (Argenta sandstone) that might be thin or missing over topographic highs in the crystalline basement^18^. It appears that Argenta formation of insufficient thickness over topographic high in the crystalline basement can no longer inhibit pressure migration into the basement faults, and those with a favorable orientation might be reactivated.

**Figure 2:**
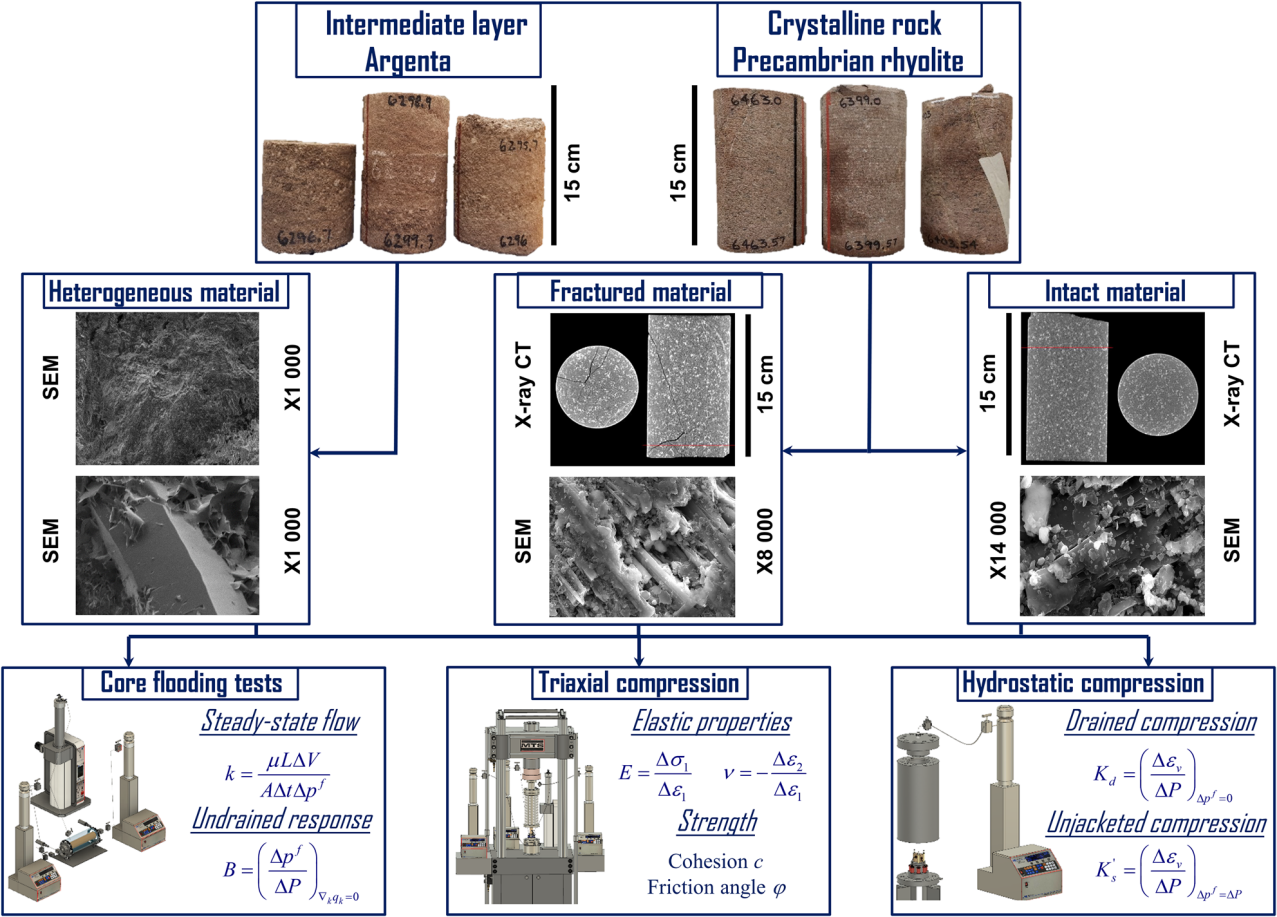
Experimental workflow for the geomechanical characterization. The studied materials are Precambrian rhyolite (crystalline basement) and Argenta sandstone (intermediate layer between the reservoir and crystalline basement). Crystalline rock specimens contain visible partially sealed fractures and intact zones without visible fractures. Testing of Argenta sandstone is conducted on the specimens of representative size to exclude effects of heterogeneity.

Many features of the induced seismicity, e.g., seismicity triggered by pore pressure decrease associated with hydrocarbon production^19^ and termination of the injection followed by continuous seismicity with the largest magnitude earthquake^20^, cannot be explained without consideration of the poroelastic effect^14^. The effects of mechanical stress state and pore pressure are coupled and cannot be considered independently. Measurements of the poroelastic properties of the material are required to address the coupling and should involve laboratory experiments in addition to the geophysical field tests^21^. This paper focuses on reporting the poromechanical properties of the Argenta sandstone and Precambrian rhyolite extracted from the T.R. McMillen #2 well located approximately 25 km southwest of the IBDP injection site. A comparison of geophysical logs for the T.R. McMillen #2 well with VW #1 at the IBDP site indicates that Argenta sandstone and Precambrian rhyolite properties are similar for these two locations. The cores of Argenta sandstone are extracted from a depth between 1919 and 1920 m, and cores of Precambrian rhyolite from a depth between 1950 and 1970 m (involved formations are encountered closer to the surface in T.R. McMillen #2 rather than they are deposited at the injection site). An essential feature of the crystalline rock is the presence of fractures, which could provide pathways for fluid migration and decrease the strength and elastic moduli of the intact material. Therefore, Precambrian rhyolite specimens are tested in fractured (with visible partially cemented fractures) and intact (without visible fractures) states (Fig. 2). Testing of the fractured rock is challenging due to the complexity of specimen preparation and the non-trivial scaling during the transition from laboratory to field scale. The Argenta sandstone is heterogeneous at small scales, so the experiments are conducted on specimens containing at least 100 times the mean grain size (0.3 mm) to minimize the effects of local features. The details about specimens’ geometry and size used for geomechanical characterization can be found in supplementary materials (Supplementary Table S1). The measured properties are implemented in fully coupled hydro-mechanical numerical code to predict the response of formations due to the CO_2_ injection and potential for induced seismicity.

## Results

### Geomechanical testing

Measurements of ultrasonic velocities, porosity, mineralogical composition, elastic properties, and strength for Precambrian rhyolite and Argenta sandstone are previously reported^12^ and are summarized along with the poroelastic properties in Supplementary Table S2 and Supplementary Fig. S2.

**Figure 3:**
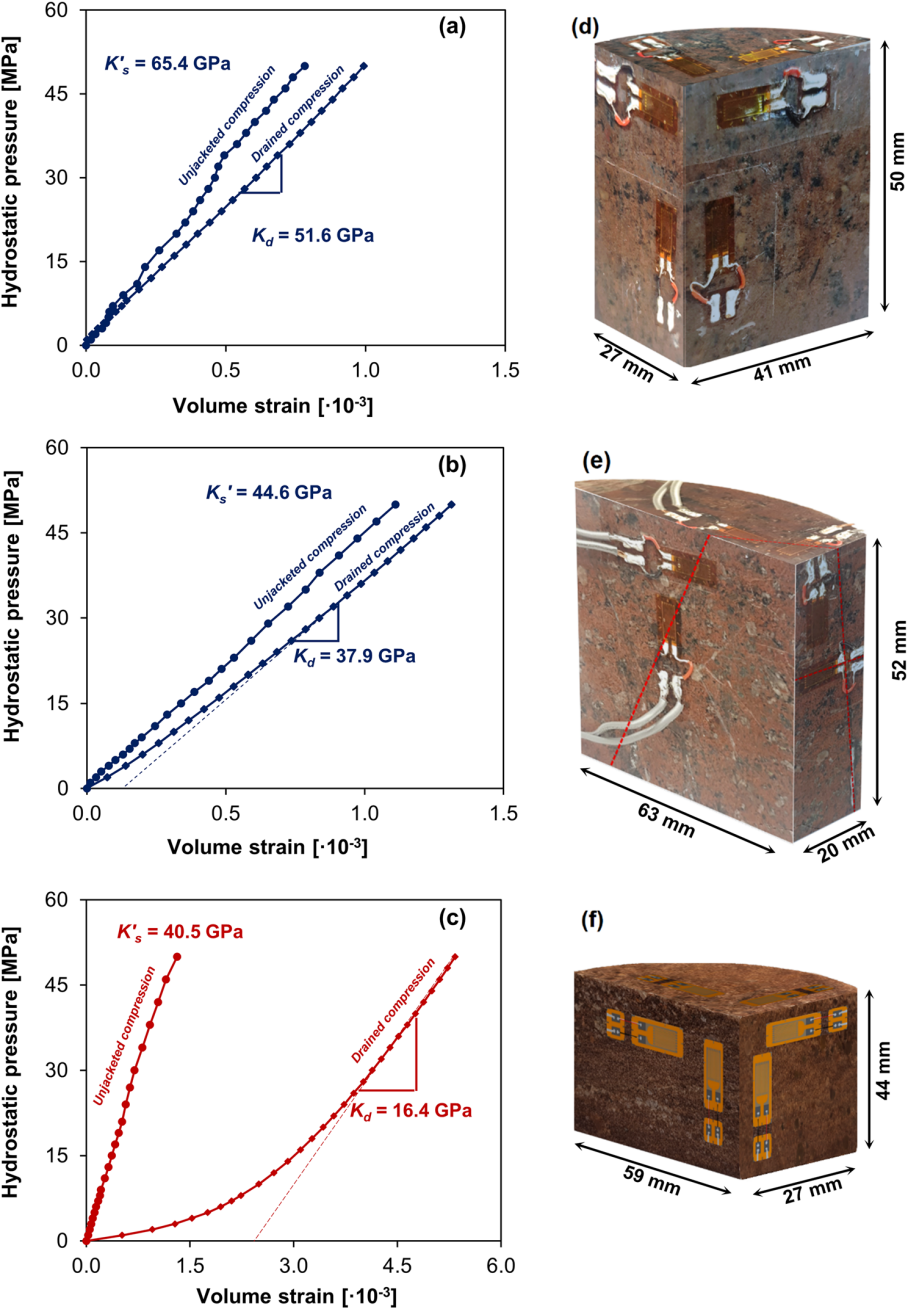
Hydrostatic pressure–volume strain diagrams from drained and unjacketed hydrostatic compression tests with (**a**) Intact Precambrian rhyolite 27 × 41 × 50 mm (**b**) Fractured Precambrian rhyolite 20 × 52 × 63 mm (fracture is highlighted with dashed line) (**c**) Argenta sandstone 27 × 44 × 59 mm. (**e**,**f**) Photographs of tested specimens with three orthogonal saw-cut surfaces.

Solid and bulk compressibilities are measured in jacketed (dry) and unjacketed hydrostatic compression tests conducted on intact Precambrian rhyolite (Fig. 3a) and Argenta sandstone (Fig. 3c) specimens, as well as on Precambrian rhyolite with visible partially cemented fractures (Fig. 3b). Photographs of tested specimens with three orthogonal saw-cut surfaces are shown in Fig. 3d–f. Due to the presence of cracks and crack-like pores, the response of Argenta sandstone and fractured Precambrian rhyolite is nonlinear at the early stages of the jacketed test. The normal strains measured during jacketed compression of Precambrian rhyolite indicate a response close to the isotropic and absence of preferential direction of microcracks (Supplementary Fig. S3). For Argenta sandstone at hydrostatic pressure below 10 MPa, the normal strain in the vertical direction is significantly higher than normal strains in two horizontal directions, making the response close to being transversely isotropic and indicating a dominantly sub-horizontal orientation of microcracks. The behavior at hydrostatic pressure higher than 10 MPa is close to isotropic (Supplementary Fig. S3). The response during the unjacketed tests is close to isotropic for all tested materials. The measured bulk and unjacketed (solid) moduli for fractured Precambrian rhyolite specimens are lower than those for the intact rock due to the presence of compressible infilling material and incomplete cement bridges. During the unjacketed test, excess pore pressure dissipation time is measured to be approximately 1 h and 24 h for Argenta sandstone and intact Precambrian rhyolite, respectively. This information is used to make an order of magnitude estimation of materials' permeability (intact Precambrian rhyolite—10^–21^ m^2^, Argenta—10^–18^ m^2^).

**Figure 4:**
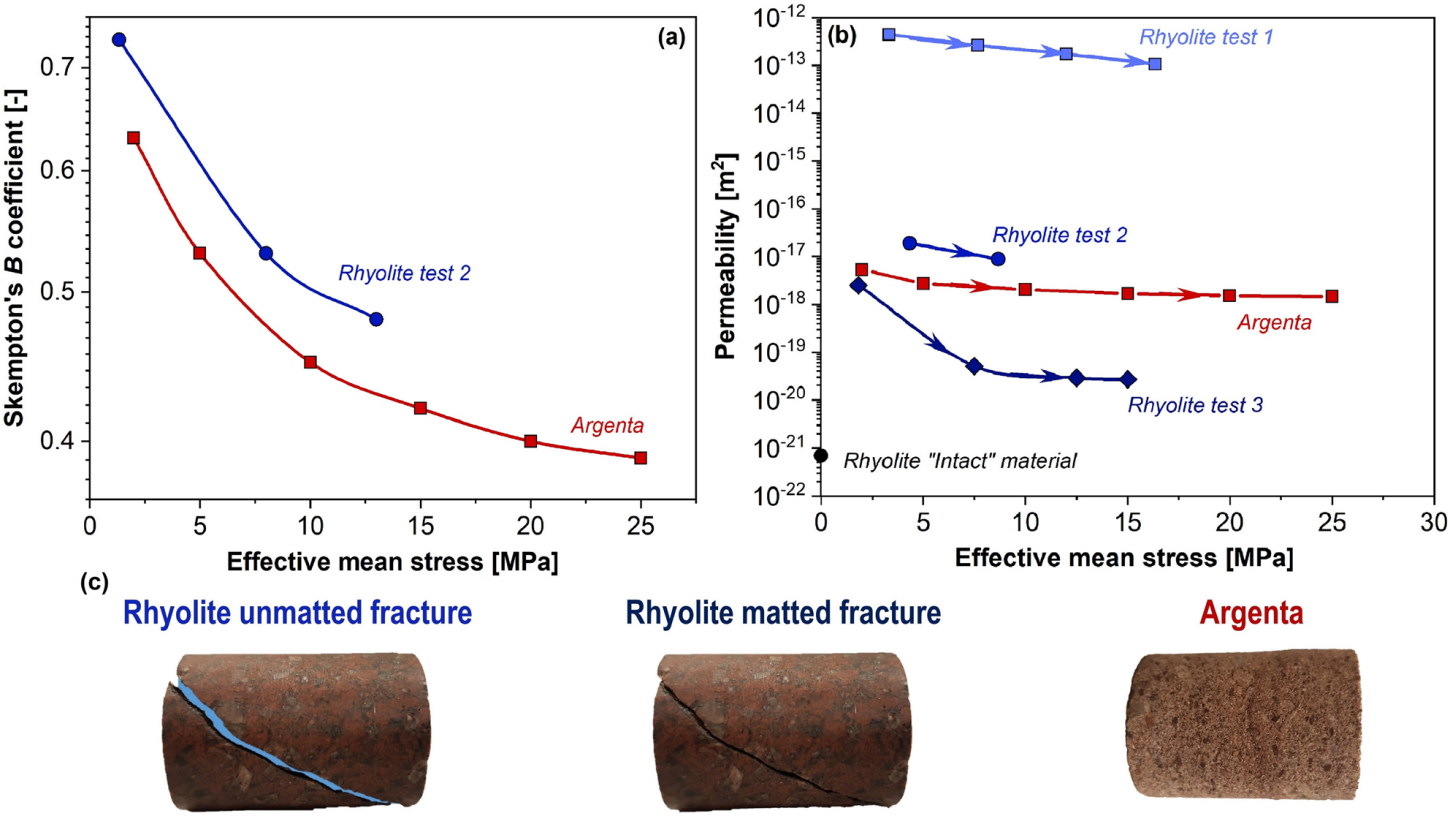
(**a**) Skempton's *B* coefficient and (**b**) permeability of fractured Precambrian rhyolite and Argenta sandstone as functions of effective mean stress. The permeability of fractured rhyolite is highly sensitive to specimen assembling and fracture opening. (**c**) Photographs of tested specimens. Tests are performed on cylindrical specimens with a diameter of 20 mm and a height of 30 mm.

Measured stress-dependent intrinsic permeability *k* and Skempton's *B* coefficient during core flooding tests with Argenta sandstone and fractured Precambrian rhyolite (with sub-vertical uncemented fracture) are shown in Fig. 4a,b. Specimens of Argenta sandstone are cored perpendicular to the bedding (along vertical in-situ direction). Photographs of tested specimens are shown in Fig. 4c. Tests on Precambrian rhyolite specimen with open fracture are repeated three times with the same specimen. However, it is expected that the fracture sides will not match each other identically after full reassembling of the experiment, so consecutive tests represent qualitatively different fracture apertures. It should be noted that fracture opening is not controlled during the test preparation and, therefore, results should be considered as a qualitative range of possible permeability of fractured specimen rather than a precise curve for permeability as a function of fracture aperture. During the first test, the fracture seems to remain partially open even at high effective mean stress providing permeability on the order of 10^–12^ m^2^. In the second test, the fracture has a different opening and measured permeability is ~ 10^–16^ m^2^. Finally, in the third test, the fracture surfaces seem to match each other closely, which results in permeability being ~ 10^–19^ m^2^ (two–three orders of magnitude higher than that of intact material) and high sensitivity to applied mean stress.

## Numerical modeling

### Motivation

**Figure 5:**
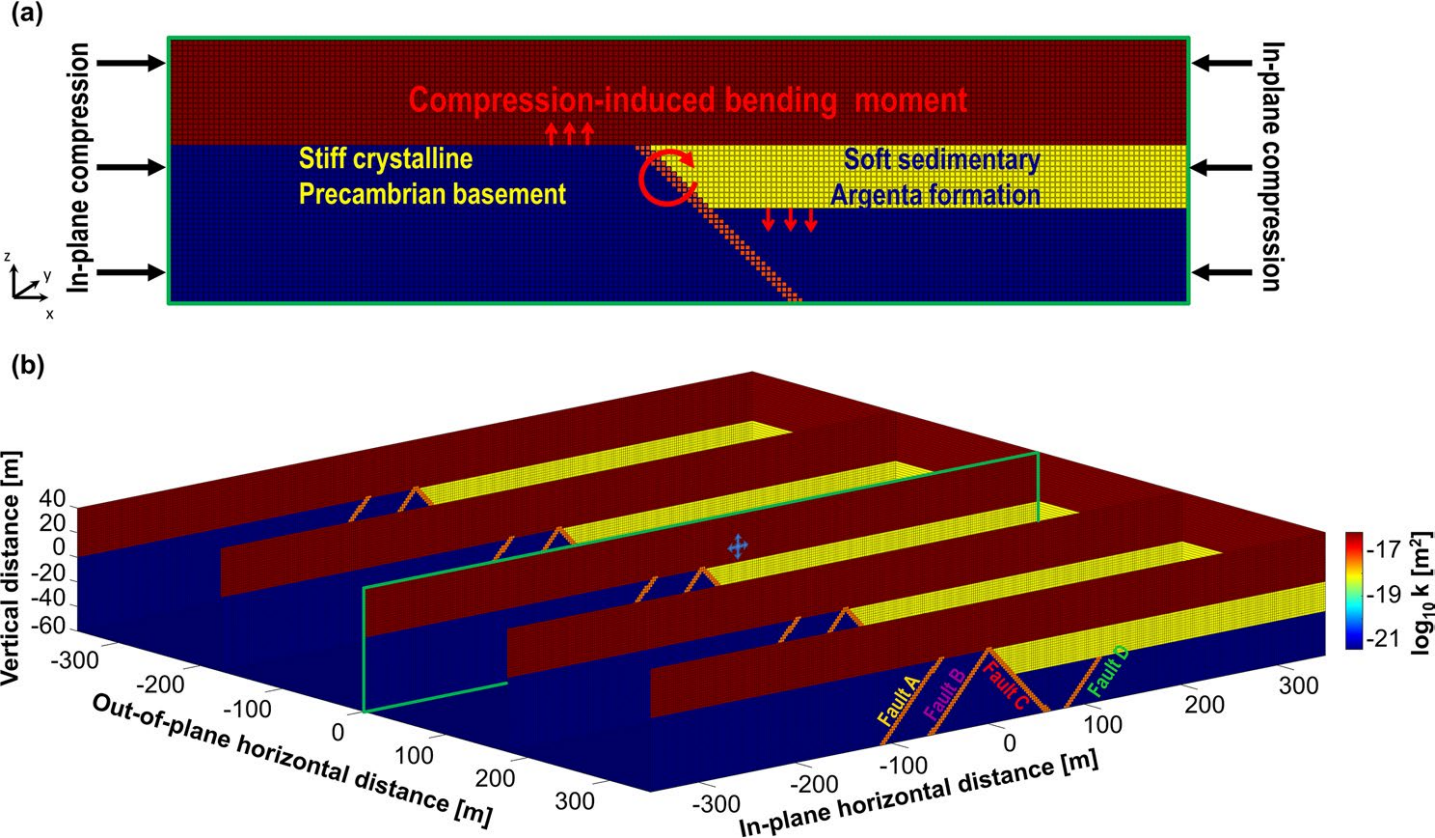
(**a**) Sketch of the mechanism of the bending moment on the interface between Argenta and Precambrian rhyolite induced by in-plane horizontal compression. (**b**) Problem layout for the numerical model with the same geometry in the out-of-plane horizontal direction that contains 5 grid points. In-plane geometry includes Mt. Simon, Argenta, Precambrian rhyolite, three high-permeable faults in the basement without an offset, and the high permeable fault with an offset. In-plane resolution is 81 grid points in the vertical direction and 567 grid points in the in-plane horizontal direction. The injection point is shown with a star, and overpressure of 2 MPa at the injection point is kept constant. Three high permeable faults (Fault A, B, C) with direct connection to the reservoir and one high permeable fault (Fault D) isolated by Argenta formation are considered.

The depth of the top part of the crystalline basement is varying over the IBDP injection site, and Argenta sandstone is mostly accumulated in the region of topographic lows of the Precambrian rhyolite with missing parts or abruptly decreased thickness over topographic highs. As a result of uneven sedimentation, juxtaposition of two different lithologies occur, creating the sub-vertical offset between Precambrian rhyolite and Argenta sandstone. Argenta formation appears to be significantly more compressible than the crystalline basement, and therefore crystalline basement could be considered as a competent layer and sedimentary Argenta—a compliant layer. Due to the high compressibility contrast, the rotational moment on the interface between the competent and compliant layers is induced by in-plane horizontal compressional stress, and this bending moment affects the state of stress in the vicinity. This mechanism is sketched in Fig. 5a.

The purpose of numerical modeling at the current stage is highlighting the role of the juxtaposed competent and compliant layers and qualitatively understanding its effect on the stress state in the vicinity. In general, the peculiarities of three-dimensional stratigraphy of the injection site will be superimposed in state of stress, making it difficult to decipher the effect of a single feature. Therefore, the simplified geometry (out-of-plane stratigraphy is assumed to remain the same) is implemented in the model and is shown in Fig. 5b. Four high permeable faults (Fig. 5b) are incorporated into numerical model. Additional details about the numerical model are provided in the methods section and supplementary materials.

### Pre-injection assessment of stress state

The state of stress at the injection interval in Mt. Simon sandstone is measured with the injection and step rate tests^13^, resulting in estimation that the minor principal stress is horizontal (*σ*_*h*_ = 34.2 MPa), the intermediate principal stress is vertical (*σ*_*v*_ = 50.8 MPa), and the major principal stress is horizontal (*σ*_*H*_ = 61.6 MPa). It is assumed that the major principal stress direction coincides with the in-plane horizontal direction, and the minor principal stress direction is out-of-plane horizontal direction, which reasonably well approximates the reported principal stress directions at the injection site^18^. The pore pressure at the injection interval is close to be hydrostatic (*p*^*f*^ = 21.8 MPa). This state of stress is favorable for strike-slip faulting regime. During the first stage of numerical modeling, boundary conditions for the model are fitted to match the known state of stress at the injection interval and predict the state of stress in underlying formations.

**Figure 6:**
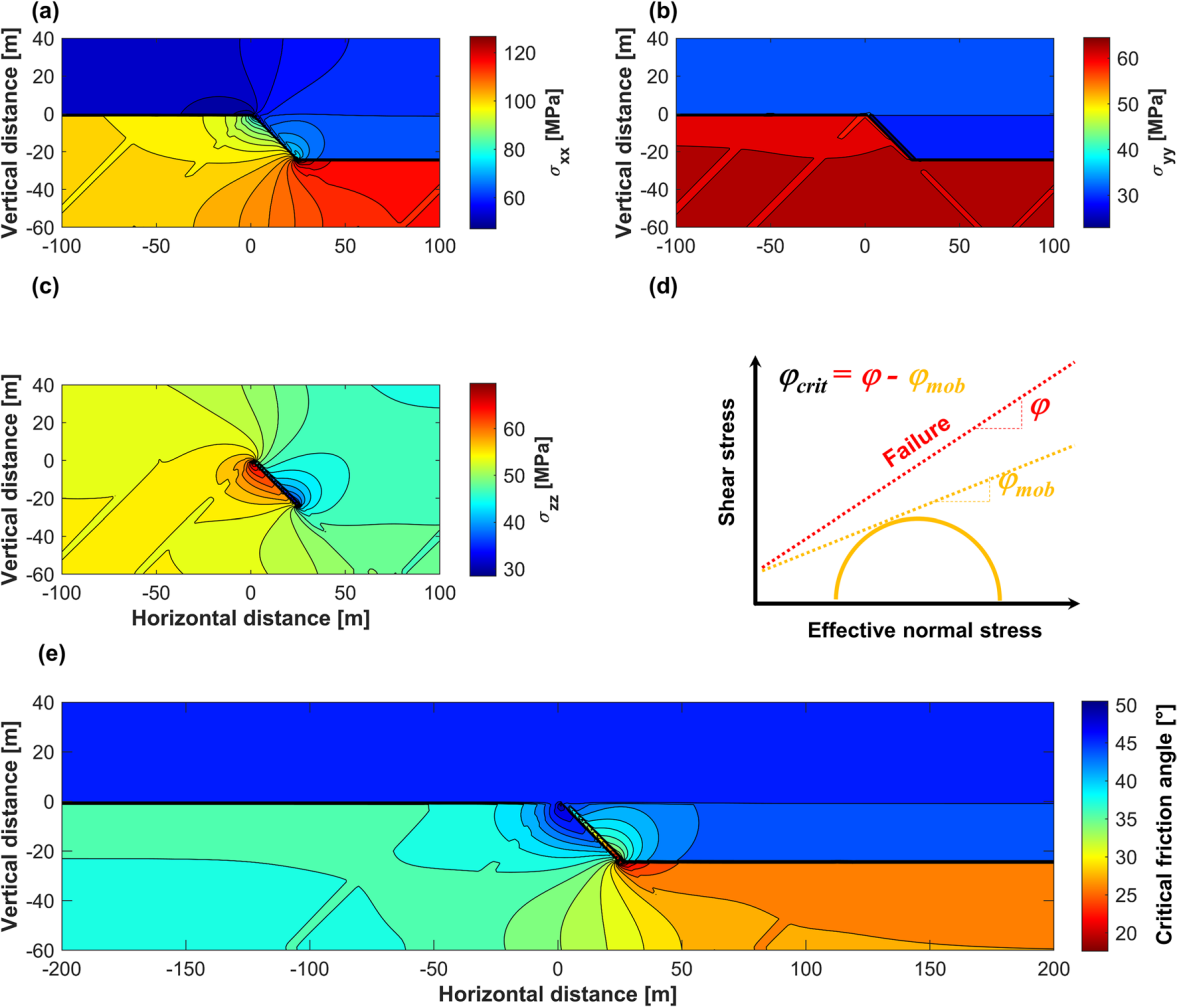
Stress state in the vicinity of the contact surface between competent and compliant layers. Contour lines of (**a**) horizontal in-plane total stress—*σ*_*xx*_, (**b**) horizontal out-of-plane total stress—*σ*_*yy*_, and (**c**) vertical total stress—*σ*_*zz*_. (**d**) Calculation of mobilized friction. (**e**) Proximity to failure in terms of the critical friction angle *φ*_*crit*_ (difference between the friction angle *φ* and mobilized friction angle *φ*_*mob*_).

The stress state near the offset in the competent layer (Fault C) is affected by the compression-induced bending moment. Contour lines of horizontal in-plane (*σ*_*xx*_), horizontal out-of-plane (*σ*_*yy*_), and vertical (*σ*_*zz*_) total stresses are shown in Fig. 6a–c. The proximity of stress state to the critical condition is evaluated in terms of the difference between friction angle *φ* and mobilized friction angle *φ*_mob_. The mobilized friction angle is calculated as a slope of the line tangential to the Mohr’s circle representing the current state of stress (Fig. 6d). The failure occurs if the mobilized friction angle *φ*_mob_ is equal to the friction angle of the material *φ* (i.e., *φ* −* φ*_mob_ approaching zero). Values of *φ*_mob_ that approach measured friction angle *φ* indicate that lower change of pore pressure is required for reactivation and region is more critically stressed. The contour lines of the *φ* −* φ*_mob_ are shown in Fig. 6e. The difference between friction and mobilized friction angles for the most part of the critically stressed region is approximately 20°. At the same time, the injection of CO_2_ for geologic carbon storage is not expected to create overpressures that would reduce *φ* − *φ*_mob_ to 0°, such that conditions favorable for significant seismic activity will not be reached.

As a result of the compression-induced bending moment, the crystalline basement to the left from the fault C is getting further from being critically stressed, while Argenta formation to the right from fault C and basement underneath Argenta are more critically stressed and require smaller change of pore pressure for reactivation. Subsequently, the offset in the competent layer results in stress state readjustment in the radius of approximately 50 m near the fault C and leads to creation of more critically stressed zones in the crystalline basement even before the injection.

### Evolution of stress state during injection

**Figure 7:**
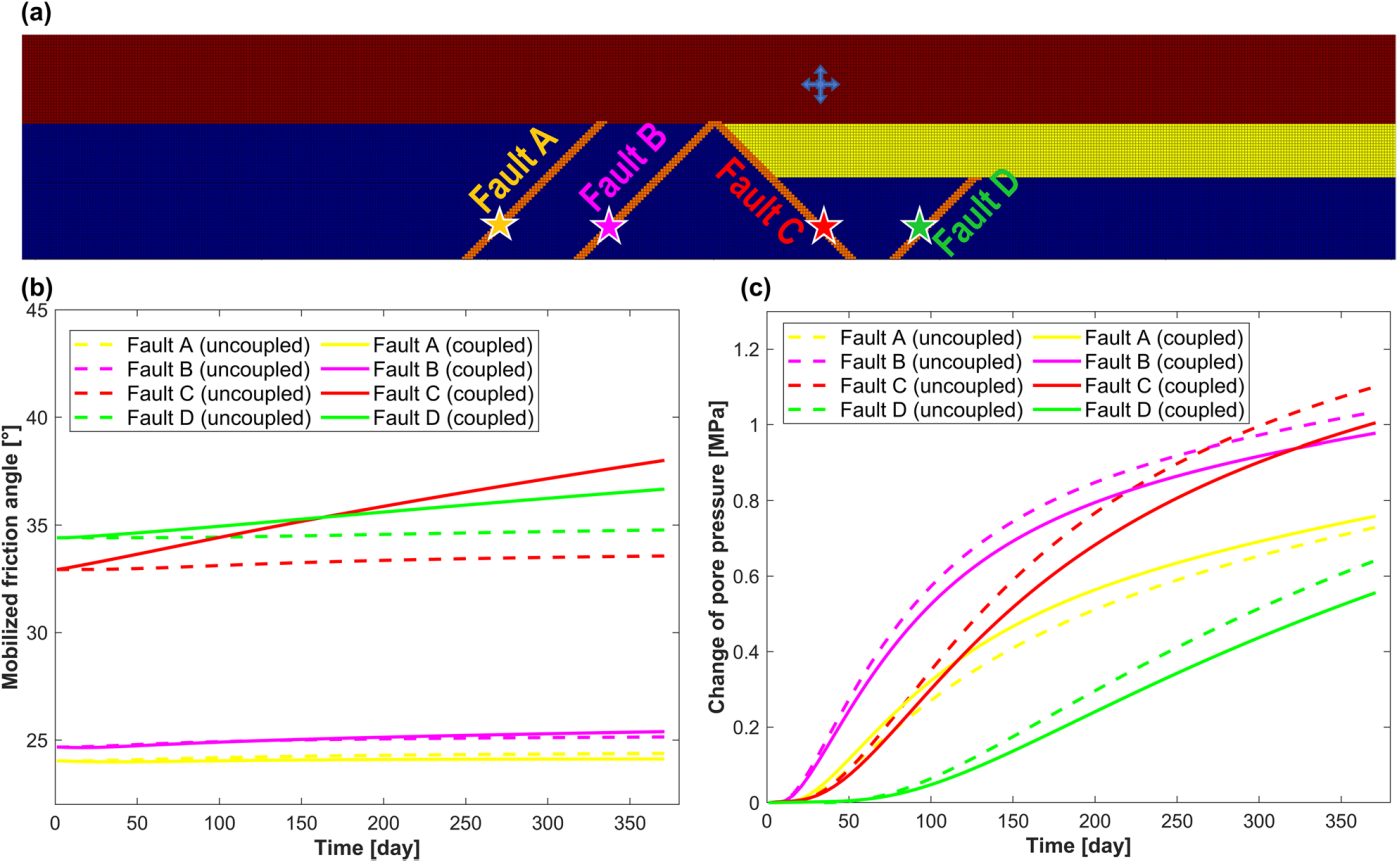
(**a**) Location of the monitoring points in the faults, (**b**) change of mobilized friction angle at monitoring points, and (**c**) change of pore pressure at monitoring points.

During the injection stage modeling, overpressure of 2 MPa is kept constant at the injection point, and the evolution of stress state is predicted. The uncoupled response is considered as a reference case, where the mechanical stresses are not changing during the injection, and only pore pressure affects the mobilized friction angle (equivalent to the shift of Mohr’s circle closer to the failure envelope). Knowledge of the poromechanical properties of formations allows consideration of the change in mechanical stresses during the injection. The mobilized friction angle is changing not only due to the change of pore pressure, but also due to the change of mechanical stresses (equivalent to change of Mohr’s circle size and its shift). The change of pore pressure with time for four monitoring points (Fig. 7a) in the basement is shown in Fig. 7c. It is observed that pore pressure change in both coupled and uncoupled models are similar. The fault D has no direct hydraulic connection to the Mt. Simon sandstone reservoir and appears to be pressurized slower compared to the faults with the hydraulic connection to the reservoir. The predicted evolution of mobilized friction angle with time for the same four monitoring points is shown in Fig. 7b. Direct (uncoupled) effect of pore pressure change does not significantly affect the stability of all faults, as shown with dashed lines in Fig. 7b. For the coupled model (shown with solid lines in Fig. 7b) the effect of injection on stability of faults C and D at monitoring points is more pronounced. Poroelastic stresses make faults C and D more critically stressed and the change of mobilized friction angle is significantly higher in case of coupled model comparing to the uncoupled one.

**Figure 8:**
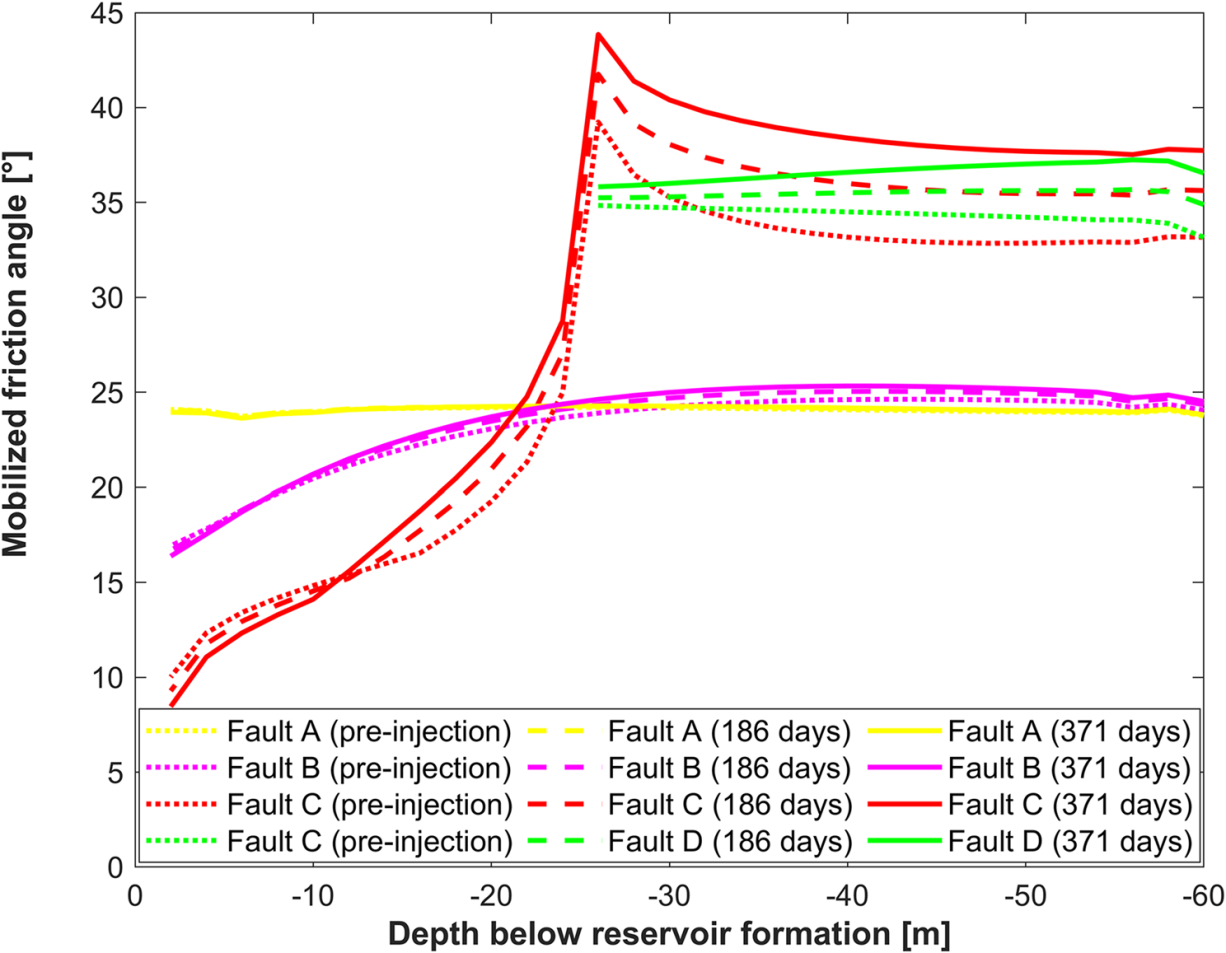
Mobilized friction angle along the faults at different moments of time. Depth of -60 m represents the deepest boundary of the model, depth of 0 m represents the interface between Mt. Simon sandstone and Precambrian/Argenta formations.

In addition, the stability along the faults is studied. In general, the distribution of mobilized friction angle is determined by the pre-injection state of stress. Injection process shifts curves to higher values of mobilized friction angle without significant effect on their shape (Fig. 8). Faults A and B and the shallow part (depth less than 25 m) of Fault C demonstrate low mobilized friction angle due to the stress redistribution near the offset in the competent layer. This region is expected to be less critically stressed and induced seismicity is unlikely to occur along these faults. Fault D and the deep (depth more than 25 m) part of Fault C are located in the region of stress concentration and therefore a higher value of mobilized friction angle is predicted. These faults are likely to be reactivated first during the injection process.

## Discussion

In this paper we report the results of laboratory geomechanical characterization of rock from the formations associated with induced microseismicity during CO_2_ injection in Illinois Basin. Poroelastic properties are measured for the crystalline basement rock in intact and fractured states, as well as for overlying low permeable sedimentary layer, which might act as a sealing for high permeable faults in the basement. Measurements of the hydromechanical properties of the intact crystalline rock are involved due to its extremely low permeability (~ 10^–21^ m^2^) leading to the total experimental time on the order of several months. On the other hand, characterization of fractured rock is non-trivial due to the complexity of specimen preparation and issues with repeatability of experiments. The laboratory-scale fractured specimens might indicate smaller aperture and higher stiffness than those at larger scale^22^. In addition, the results of permeability measurements and stress-permeability relation are also known to be size-dependent^10^. Finally, the laboratory tests are usually conducted on specimens with one or several fractures, while the fracture network should be considered for the in-situ conditions^23^. All of these challenges need to be properly addressed before the implementation of laboratory measured properties in the reservoir-scale numerical models. The conducted permeability measurements reported for the fractured rock are highly sensitive to the experimental setup. Depending on the fracture aperture, the permeability could vary up to seven orders of magnitude for the same tested specimen and be up to nine orders of magnitude higher than the permeability of the intact material.

Comparison between the prediction with uncoupled and fully hydro-mechanically coupled numerical models suggests that poroelastic stresses are the main mechanism for destabilization of the faults considered in this study. It should be noted that the absolute value and sign (increase or decrease) of poroelastic stresses depends on the fault location and orientation. The simplified model geometry does not allow to confidently conclude that poroelastic stresses are the main triggering mechanism of induced microseismicity at IBDP. However, presented results highlight the need for developing high-resolution three-dimensional hydro-mechanically coupled models allowing to analyze the subsurface injection and potential seismicity.

Presence of a low permeable Argenta formation between the injection interval and critically stressed basement prevents the pressurization of high-permeable faults. Basal sealing layers similar to Argenta formation might prevent reactivation of faults due to the reduced direct pore pressure transfer effect. This result is similar to the predicted reduction of induced earthquakes potential by the bottom seals with low permeability or high storativity^15^. In contrast to the first stage of CO_2_ injection at the Decatur site analyzed in this paper, the second stage injection was performed 100 m shallower (above the intraformational seal) in the Mt. Simon sandstone reservoir, and it caused a significant reduction of the observed microseismic events in the basement^18^. The conducted numerical modeling predicts that Argenta formation restricts the connection of fault D with the injection reservoir preventing the pressurization of this fault. Despite of the direct pore pressure transfer effect inhibited by the basal seal, the coupled hydromechanical effect needs to be considered, since the stability of the faults is affected not only by pore pressure variations, but also by the changes in the mechanical stresses due to the poroelastic effect^14^. In addition to the poroelastic stresses induced during the injection, the event triggering mechanism might be important for microseismic response^24^ and needs to be addressed in further studies.

Observed microseismicity during CO_2_ injection at IBDP does not directly correlate with the propagation of the pressure front and has a tendency for clustering^17^. Clusters of the microseismic events might be associated not only with locally weak fractured material but also with features of the stress state governed by the stratigraphy. The offset in stiff competent crystalline basement (Precambrian rhyolite formation) will require a rotation moment to balance the in-plane compressional stress. This bending moment will cause stress redistribution near the stratigraphic features. Therefore, knowledge of local stratigraphy of injection site combined with results of laboratory geomechanical characterization might be utilized to predict critically stressed zones before the injection. We believe that juxtaposition of the competent and compliant layers is capable to create zones which are more critically stressed before the injection, therefore affecting the location of observed clusters of microseismicity. It is suggested that even though faults in the basement provide pathways for the pressure migration, the main mechanism for the triggered microseismicity is associated with the change of mechanical stresses due to the poroelastic effect. The assumption that geometry is not changing in the out-of-plane horizontal direction is an oversimplification of the in-situ conditions. Therefore, the direct comparison between observed microseismicity and predicted critically stressed zones seems to be premature. Only high-resolution three-dimensional model can accurately predict all critically stressed zones and verify that poroelastic stresses are the main triggering mechanism for induced microseismicity.

The general recommendation is that thorough geomechanical characterization of rock formations needs to be combined with the proper description of the injection site topography. It allows to estimate the pre-injection stress state which determines areas more prone for induced microseismic response. Representative three-dimensional numerical models are required to calculate the evolution of pore pressure and mechanical stresses during the injection and determine the particular triggering mechanism for induced microseismicity during large scale CO_2_ injection.

## Methods

### Hydrostatic compression

The jacketed *K*_*d*_ (dry or drained) and unjacketed *K*′_*s*_ bulk moduli of rock are accurately measured during the hydrostatic compression test^25^. The hydrostatic pressure is applied with oil and is controlled by a hydraulic pump. A specimen with three orthogonal saw-cut surfaces is instrumented with strain rosettes in three perpendicular directions, such that the volume strain *ε*_*v*_ is calculated as the sum of three orthogonal normal strains. The specimen is covered with a polyurethane membrane, submerged in the hydraulic oil, and the material's response due to hydrostatic loading is recorded. Since the membrane prevents any fluid penetration, the change of pore pressure during the loading is zero, and the drained (dry) bulk modulus *K*_*d*_ is measured. After the jacketed test, the membrane is removed, and the procedure is repeated under the unjacketed condition (the change of pore pressure Δ*p*^*f*^ is equal to the change of total mean stress Δ*P*) to report the corresponding unjacketed bulk modulus *K'*_*s*_, sometimes referred to as modulus of the solid constituents. Consequently, the Biot coefficient *α* is calculated as *α* = 1−*K*_*d*_/*K'*_*s*_.

During the unjacketed test, the pore fluid pressure must equilibrate with the hydrostatic pressure after each loading step. Dissipation time of excess pore pressure depends on the material permeability, and for low permeable rock might take up to several days, leading to total experimental time on the order of months. Dissipation time might be utilized for the first-order estimation of rock permeability with the knowledge of other material properties.

1 $$k \approx D\mu \phi \left( {\frac{1}{{K_{f} }} + \frac{\alpha }{{\phi K_{d} }}} \right).$$

Here *μ* is the dynamic viscosity of the fluid, in our case—hydraulic oil (*μ*_*oil*_ = *0.07* Pa·s), and *K*_*f*_ is the bulk modulus of pore fluid (*K*_*oil*_ = 1.3 GPa). The hydraulic diffusivity *D* is proportional to squared characteristic length *l* and inversely proportional to the diffusion time *t* (*D* ~ *l*^*2*^*/4t* for double drainage boundary condition).

### Core flooding

The core flooding device is used for measurements of permeability and undrained response^26^. Rock specimen is placed between two platens and the upstream and downstream pore pressure, as well as the lateral oil pressure are controlled with the hydraulic pumps. All the pumps can be operated in pressure, volume, and flow control modes. Pressure control mode is utilized for imposing drained boundary condition, where pore pressure in the specimen is equilibrated with the fluid pressure in the pump. Undrained boundary condition means that mass of fluid in the pore space remains constant and is imposed by closing valves between the upstream/downstream pump and the core holder, while the upstream/downstream fluid pressure is accurately measured via pressure transducers.

A back pressure saturation technique is utilized to achieve full saturation by forcing any gas in the pore space to dissolve into the pore water^27^. Measurements of the Skempton's *B* coefficient are performed at gradually increasing back pressures while keeping the Terzaghi effective mean stress *P'* = *P − p*^*f*^ constant. When constant *B* value independent of the magnitude of the back pressure at fixed effective mean stress is achieved—the full saturation is assumed and the intrinsic permeability is measured with the steady-state fluid flow technique.

### Numerical modeling

The implemented model is based on consideration of a two-phase continuum (solid and fluid phases) and numerically solving Biot equations with the finite difference method at each grid point. The equations are written in a symmetric form by separating volumetric and deviatoric parts of the stress tensor^28,29^:

2 $$\left( \begin{gathered} \frac{\partial P}{{\partial t}} \hfill \\ \frac{{\partial p^{f} }}{\partial t} \hfill \\ \end{gathered} \right) = - K_{u} \left( {\begin{array}{*{20}c} 1 & B \\ B & {B/\alpha } \\ \end{array} } \right)\left( \begin{gathered} \nabla_{k} v_{k}^{s} \hfill \\ \nabla_{k} q_{k} \hfill \\ \end{gathered} \right),$$

3 $$\frac{{\partial \tau_{ij} }}{\partial t} = 2G\left( {\frac{1}{2}\left( {\nabla_{i} v_{j}^{s} + \nabla_{j} v_{i}^{s} } \right) - \frac{1}{3}\nabla_{k} v_{k}^{s} \delta_{ij} } \right),$$

where *P* is the total mean stress, *p*^*f*^ is the fluid pressure, *τ*_*ij*_ is the total stress deviator, *K*_*u*_ is the undrained bulk modulus, *G* is the shear modulus, *B* is the Skempton’s coefficient, *α* is the Biot’s coefficient, *v*^*s*^ is the velocity of solid phase, *q* is the Darcy’s flux, and *δ*_*ij*_ is the Kronecker delta.

If non-diagonal components of matrix in Eq. (2) are equal to zero, the model is uncoupled. In this case, fluid pressure and mechanical stresses are independent of each other and only pore pressure is changing during the injection. Nonzero components of the matrix provide hydro-mechanical coupling, which results in pore pressure and mechanical stresses changing during the injection. These additional mechanical stresses occurred due to the change of pore pressure are being referred as poroelastic stresses.

The conservation of linear momentum (Newton’s Second law) can be also written in a symmetric form^28,29^:

4 $$\left( \begin{gathered} \nabla_{j} \left( { - P\delta_{ij} + \tau_{ij} } \right) \hfill \\ \frac{\mu }{k}q_{i} + \nabla_{i} p^{f} \hfill \\ \end{gathered} \right) = \left( {\begin{array}{*{20}c} {\rho_{t} } & { - \rho_{f} } \\ { - \rho_{f} } & {\rho_{a} } \\ \end{array} } \right)\left( \begin{gathered} \frac{{\partial v_{i}^{s} }}{\partial t} \hfill \\ - \frac{{\partial q_{i} }}{\partial t} \hfill \\ \end{gathered} \right),$$

where *μ* is the dynamic fluid viscosity and *k* is the intrinsic permeability. The accelerated pseudo-transient iterative method is utilized to achieve an equilibrium state of stress at each timestep^30^. The right-hand side of the Eq. (4) is equal to zero at quasistatic equilibrium stress of state (since ∂*v*_*i*_*/*∂*t* = 0, ∂*q*_*i*_*/*∂*t* = 0). Therefore, components of the density matrix (*ρ*_*t*_, *ρ*_*f*_, *ρ*_*a*_) affect only the number of iterations to calculate the equilibrium state of stress and not the stress state itself.

The continuum is discretized with a staggered space–time grid^31^ and the example of a grid is shown in Supplementary Fig. S1 Equations (2–4) are solved numerically at each grid point with the finite difference method. The damping of the velocity of the solid phase is used to provide a mechanism for elastic wave attenuation and calculate the quasistatic distribution of the solid phase velocity *v*^*s*^ and Darcy’s flux *q* at the moment of time *t* based on Eq. (4). After that, velocities are substituted into Eqs. (2, 3) to calculate the stress state and pore pressure at *t* + Δ*t*. The timestep Δ*t* is a function of the mesh spacing Δ*x*, Δ*y*, Δ*z* and is chosen to provide stability of the numerical scheme of poroelastic equations^29^. The numerical simulation and graphics routine are realized in Matlab©.

Measured laboratory properties are summarized in Supplementary Table S2 and are implemented in the numerical model. Model is simplified by the assumption that out-of-plane geometry remains the same, and therefore low resolution (5 grid points) is utilized for out-of-plane direction. The physical size of the model is 700 × 700 × 100 m. In-plane resolution is 81 grid points in the vertical direction and 567 grid points in the horizontal one. The boundary conditions for displacement are applied to match measured total stresses and pore pressure at the injection point: *σ*_*3*_ = *σ*_*h*_ = 34.2 MPa, *σ*_*2*_ = *σ*_*v*_ = 50.8 MPa, *σ*_*1*_ = *σ*_*H*_ = 61.6 MPa, and *p*^*f*^ = 21.8 MPa. After reaching the mechanical equilibrium and hydrostatic pore pressure distribution in the model, the mobilized friction angle is calculated and proximity to failure is shown in terms of the difference between the friction angle and mobilized friction angle (difference equal to zero means achieving the failure condition). In the next stage, constant overpressure of 2 MPa is maintained at the injection point and change of pore pressure and mechanical stresses is monitored in each fault to study the potential triggering mechanism for induced microseismic response.

## Supplementary Information

Supplementary Information.

## Data Availability

Data associated with this research are available from the corresponding author per request.
