# Supplementary material for: Hydromechanical impact of basement rock on injection-induced seismicity in Illinois Basin
Source: Sci Rep. 2022 Sep 19;12:15639. doi: 10.1038/s41598-022-19775-4 (PMC9485228; doi:10.1038/s41598-022-19775-4)
Supplement: Supplementary file 1 — Supplementary Information. [file 41598_2022_19775_MOESM1_ESM.pdf]

Supplementary information for

Hydromechanical impact of basement rock on injection-induced seismicity in Illinois Basin

Nikita Bondarenko*[1], Yury Podladchikov[2] and Roman Makhnenko[1]

[1]University of Illinois at Urbana-Champaign, Department of Civil and Environmental Engineering, Urbana, Illinois, USA. *E-mail: nikitab3@illinois.edu

[2]University of Lausanne, Institute of Earth Sciences, Lausanne, Switzerland.

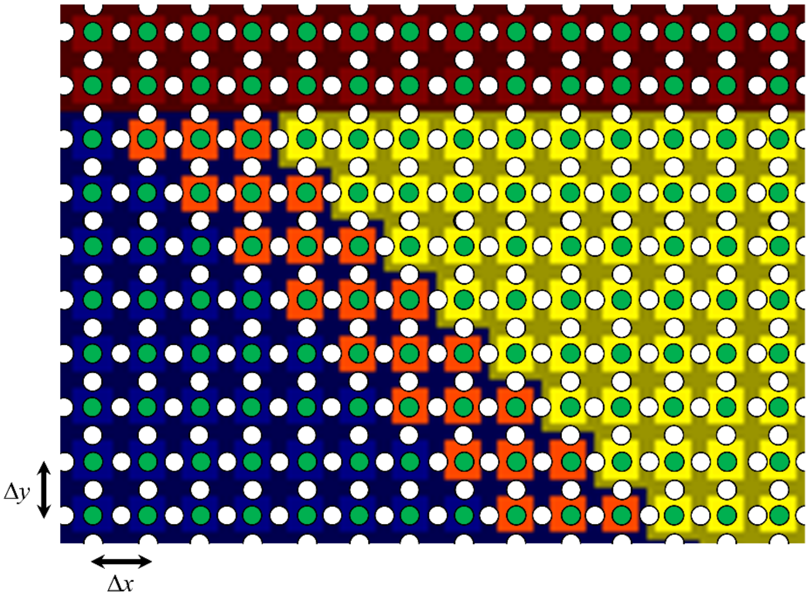

Figure S1. Example of the staggered space grid. Equation (5) is solved numerically at each grid point shown with the white circles to calculate the velocity of the solid phase v^s and Darcy's flux q at the moment of time t. After that velocity of the solid phase and Darcy's flux are substituted into Equations (3, 4) to calculate the mechanical stresses and pore pressure at each grid point shown with the green circles at the moment of time $t + \Delta t$.

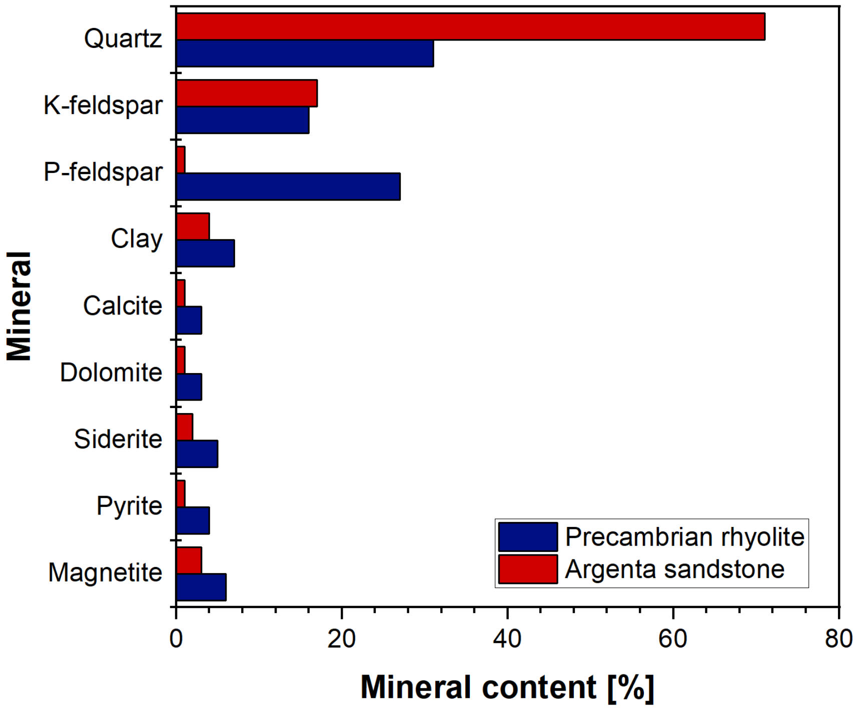

Figure S2. Mineralogical composition of Precambrian rhyolite and Argenta sandstone based on the petrographic analysis.

The hydrostatic compression tests are firstly conducted on specimens covered with polyurethane jacket to measure drained bulk modulus K, and after that, the jacket is removed and unjacketed bulk modulus K'_s is measured.

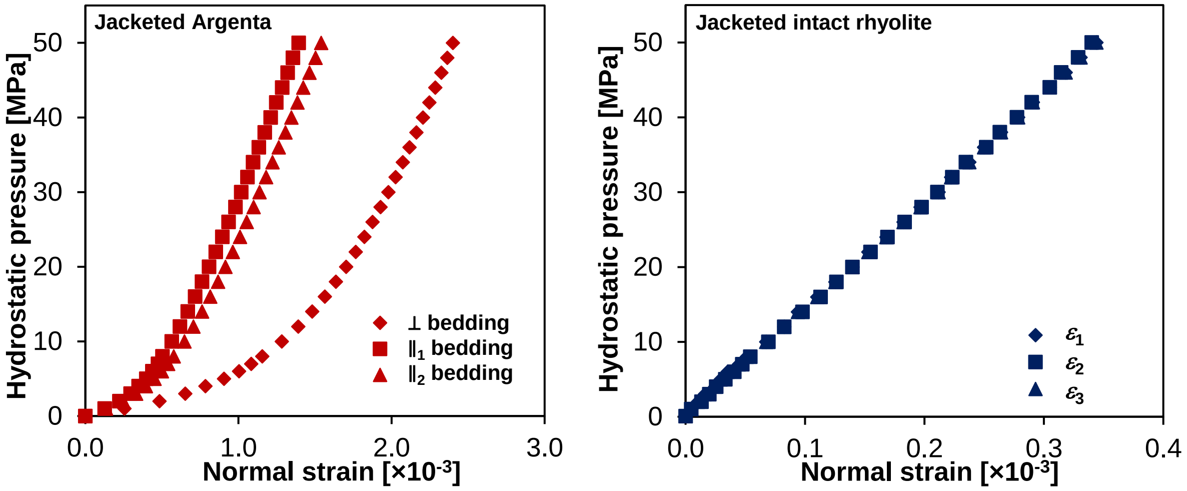

Figure S3. Measured normal strains during jacketed compression of Argenta sandstone and intact Precambrian rhyolite. The response of Argenta sandstone is close to transversely isotropic and response of intact Precambrian rhyolite is isotropic. The bedding planes in Argenta are sub-horizontal, normal strains are measured in two directions parallel to the bedding (horizontal in-situ directions), and one perpendicular to the bedding (vertical is-situ direction).

Table S1. Summary of the experimental procedures used for the geomechanical characterization

<table>
<tr><td>Property</td><td>Precambrian rhyolite (fractured)</td><td>Precambrian rhyolite (intact)</td><td>Argenta sandstone</td><td>Mt. Simon sandstone</td></tr>
<tr><td rowspan="2">Unjacketed bulk modulus, K'_s [GPa]
Drained bulk modulus, K_d [GPa]</td><td colspan="3">Hydrostatic compression tests</td><td rowspan="6">Properties are adapted from [2, 3] and typical values for reservoir rock</td></tr>
<tr><td>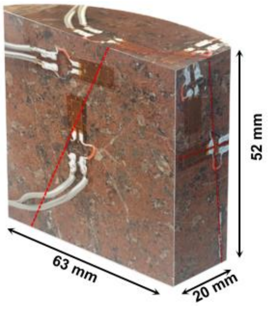
</td><td>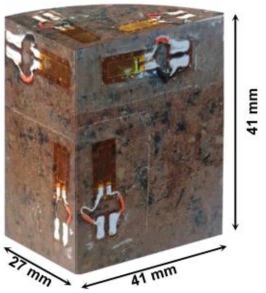
</td><td>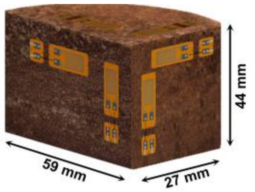
</td></tr>
<tr><td rowspan="2">Poisson's ratio, ν [-]
Shear modulus, G [GPa]
Cohesion, c [MPa]
Friction angle, φ [°]</td><td colspan="3">Conventional triaxial compression tests</td></tr>
<tr><td>N/A</td><td colspan="2">Cylindrical specimens:
d = 50.8 mm and h = 101.6 mm
d = 30.0 mm and h = 60.0 mm
Details could be found in [1]</td></tr>
<tr><td rowspan="2">Intrinsic permeability, k [m^2]
Skempton's coefficient, B [-]</td><td colspan="3">Core flooding tests</td></tr>
<tr><td>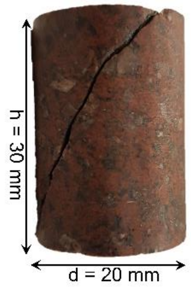
</td><td>N/A</td><td>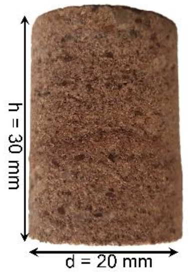
</td></tr>
</table>

Table S2. Summary of the measured material properties implemented in the numerical model

Property	**Precambrian rhyolite (fractured)**	**Precambrian rhyolite (intact)**	**Argenta sandstone**	**Mt. Simon sandstone**
Unjacketed bulk modulus, K'_s [GPa]	44.6	65.4	40.5	36.7
Drained bulk modulus, K_d [GPa]	37.9	51.6	16.4	18.0
Undrained bulk modulus, K_u [GPa]	42.7*	57.7*	21.5*	26.9*
Biot coefficient, α [-]	0.15*	0.21*	0.60*	0.51*
Pore fluid bulk modulus, K_f [GPa]	2.1			
Poisson's ratio, ν [-]	0.35	0.22	0.22	0.23
Shear modulus, G [GPa]	30.7	45.0	27.9	24.2
Intrinsic permeability, k [m^2]	~10^{-18}	~10^{-21} **	~10^{-18}	~10^{-16}
Porosity, ϕ [-]	0.020	0.005	0.138	0.115
Skempton's coefficient, B [-]	0.75	0.5*	0.40	0.65
Cohesion, c [MPa]	0	0	11.2	25.5
Friction angle, φ [°]	61	61	42	47

* Calculated based on the other poroelastic properties

** Estimation from the transient process during unjacketed hydrostatic compression

$$\alpha = 1 - \frac{K_d}{K_s^{'}} \quad \text{(s1)}$$

$$K_u = \frac{K_d}{(1-\alpha B)} \quad \text{(s2)}$$

$$B = \frac{\alpha}{\alpha + \phi K_d \left(\frac{1}{K_f} - \frac{1}{K_s^{''}} \right)} \quad \text{(s3)}$$

The unjacketed pore modulus $K_s^{''}$ is assumed to be equal to the unjacketed bulk modulus $K_s^{'}$

REFERENCES

1. Bondarenko, N., Williams-Stroud, S., Freiburg, J. & Makhnenko, R. Geomechanical aspects of induced microseismicity during CO_2 injection in Illinois Basin. *The Leading Edge*, **40**(11), 823-830 (2021).
2. Freiburg, J.T., Ritzi, R.W., & Kehoe, K.S. Depositional and diagenetic controls on anomalously high porosity within a deeply buried CO2 storage reservoir - the Cambrian Mt. Simon Sandstone, Illinois Basin, USA. *Int. J. Greenhouse Gas Control*, 55, 42–54 (2016).
3. Bauer, R.A., Carney, M., & Finley, R.J. Overview of microseismic response to CO_2 injection into the Mt Simon saline reservoir at the Illinois Basin-Decatur project. *Int. J. Greenhouse Gas Control*, 54(1), 378-388 (2016).
